# Water as a “glue”: Elasticity-enhanced wet attachment of biomimetic microcup structures

**DOI:** 10.1126/sciadv.abm9341

**Published:** 2022-03-23

**Authors:** Yue Wang, Zhengwei Li, Mohamed Elhebeary, René Hensel, Eduard Arzt, M. Taher A. Saif

**Affiliations:** 1 INM-Leibniz Institute for New Materials, Saarbrücken, Germany.; 2 Mechanical Science and Engineering, University of Illinois at Urbana-Champaign, Urbana, IL 61822, USA.; 3 Saarland University, Materials Science and Engineering, Saarbrücken, Germany.

## Abstract

Octopus, clingfish, and larva use soft cups to attach to surfaces under water. Recently, various bioinspired cups have been engineered. However, the mechanisms of their attachment and detachment remain elusive. Using a novel microcup, fabricated by two-photon lithography, coupled with in situ pressure sensor and observation cameras, we reveal the detailed nature of its attachment/detachment under water. It involves elasticity-enhanced hydrodynamics generating “self-sealing” and high suction at the cup-substrate interface, converting water into “glue.” Detachment is mediated by seal breaking. Three distinct mechanisms of breaking are identified, including elastic buckling of the cup rim. A mathematical model describes the interplay between the attachment/detachment process, geometry, elasto-hydrodynamics, and cup retraction speed. If the speed is too slow, then the octopus cannot attach; if the tide is too gentle for the larva, then water cannot serve as a glue. The concept of “water glue” can innovate underwater transport and manufacturing strategies.

## Teaser

Under-water soft cups form strong attachment with solid surfaces upon retraction by generating large transient suction.

## INTRODUCTION

Water is usually not considered a “glue.” It tends to attenuate intermolecular forces and prevents close contact between two solid bodies (*1*). Hydrophilic surfaces are separated by several monolayers of water (~1 nm) (*2*, *3*), well beyond the typical range of van der Waals interactions (<0.6 nm) (*1*). The average gap is expected to be even higher because of surface asperities and roughness. These limitations pose a substantial challenge for reversible attachment under wet conditions. These attachments are required in various medical and industrial applications such medical patches (*4*), tissue engineering, or underwater soft robotics (*5*, *6*). Microfibrillar designs, which produce reliable adhesion in the dry state (*7*), are useful under wet conditions only after displacing water from the contact zone: Tried strategies are hydrophobic designs (*8*, *9*), direct chemical bonding (*10*), or dynamic bonds in tough hydrogels (*11*). These solutions limit the variability of the counter surface and suffer from drawbacks related to durability or swelling.

**Fig. 1.**
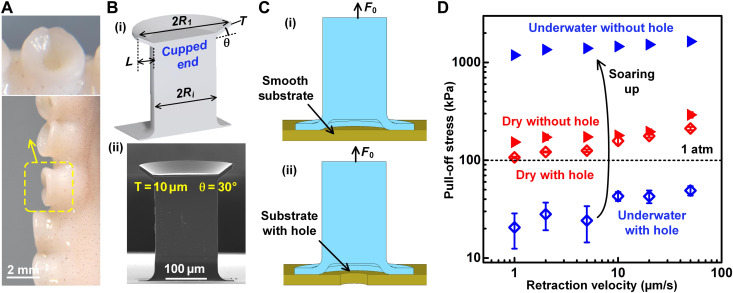
Bioinspiration and its implementation in underwater attachment. (**A**) Suction cups of live octopus (photo credit: Eduard Arzt, author). (**B**) Cupped microstructure used for underwater attachment tests: (i) schematic and (ii) scanning electron microscope image. (**C**) Illustrations of microcup tested against a silicon wafer with and without a microhole, which is aligned with the center of the cup, and (**D**) pull-off stress from tests in air and under water against the wafer with/without a microhole at different velocities.

We propose here to reverse the role of water and turn it into a glue. Several aquatic animals such as octopus (Fig. 1A), clingfish, and remoras use suction cups to attach to surfaces for locomotion (*12*–*15*). Suction cups do not create attachment by intermolecular adhesion but by a differential between inside and outside pressure; this is the reason why the usage of “attachment” is preferred throughout the text. Inspired by nature, several suction cups have been designed for underwater application. Attachment stresses of underwater suction cups have been found to be unexpectedly high, up to 1 MPa [~0.8 MPa for octopus cups (*16*, *17*) and ~1 MPa for engineered cups (*18*, *19*)], an order of magnitude higher than atmospheric pressure (0.1 MPa), which limits dry suction. The limit for wet conditions is posed by the cavitation pressure of pure water, which ranges from 17 to 26 MPa (*20*) to 140 MPa (*21*). Thus, suction cups should be a much more powerful attachment strategy in water than in air. Capillarity mechanisms require three phases—solid, liquid, and air/vapor (*22*, *23*)—and are not considered here.

The mechanism of bioinspired underwater suction is, however, largely underestimated as a possible engineering solution. The reason is that, in contrast to suction cups in air (*24*), mechanistic experiments in water are almost totally lacking in the literature. Hence, the origin of strong attachment in underwater cups remains elusive. If suction is the primary source of strong attachment, then what limits its value? What is the mechanism of detachment or failure of the cup? In addition, how will the performance scale with size, especially down to micro dimensions? Understanding such mechanism of operation quantitatively will allow to rationally design cups for underwater use, for both household and industrial applications.

**Fig. 2.**
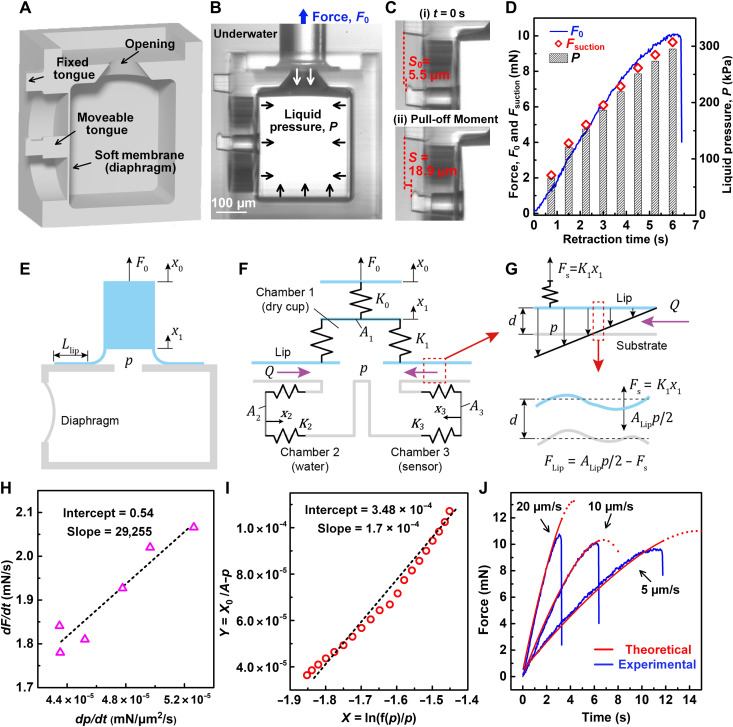
Pull-off experiments with built-in micro pressure sensor. (**A**) Schematic of substrate with a hole and a soft membrane acting as a pressure sensor; (**B**) in situ microscopy image of the suction cup and sensor; (**C**) membrane bending images at the start of retraction and near pull-off. (**D**) Attachment force *F*_0_ recorded from load cell, liquid pressure *p* calculated from membrane deflection, and suction force *F*_suction_ estimated on the basis of liquid pressure *p*. (**E**) Schematic of underwater experiment with in situ pressure measurement; (**F**) simplified mechanical model; (**G**) suction pressure distribution along the contact lip and forces acting on the lip; (**H**) rate of change of force and pressure; (**I**) comparison between the theoretical model predictions and experimental data. (**J**) Evolution of force with time at different retraction speeds, model versus experiment.

The present paper proposes the first design of a miniaturized suction cup that sheds light on the nature of the attachment mechanism. Using advanced two-photon lithography and molding process at submillimeter scale (*25*), we fabricate polyurethane microcups consisting of a stalk and a conical lip (Fig. 1B). The cup is inverted and pressed against a flat substrate (Fig. 1C). During retraction at a prescribed constant speed, ${\overset{\cdot }{x}}_{0}$, we optically observe the contact phenomena. In addition, we develop a novel built-in pressure sensor (Fig. 2, A and B) to measure the pressure inside the cup during retraction in situ, in microscale. With the help of a mathematical model, the results are analyzed to create a deeper understanding of attachment in a wet environment.

## RESULTS

### Role of van der Waals force in underwater suction cups

To explore the role of van der Waals force in microcups, we carry out tests both in air and in water using a cup with stalk radius *R_i_* = 80 μm and cup radius *R*_1_ = 120 μm, cap thickness of 10 μm, and tilt angle θ of 30° (Fig. 1B). A double-sided polished silicon wafer was used as the substrate. A 60-μm square hole was etched through the wafer for some of the substrates. The opening allows fluid (air or water) flow during retraction, and suction is reduced or cannot develop at all. The cup was pressed against the substrate, with and without the hole, with a preload of 2 mN, holding for 5 s, and then retracted with velocity from 1 to 50 μm/s (Fig. 1D). These test results (Fig. 1D) reveal the following:

1) In air, the pull-off stress is similar for substrates with and without the hole, implying that suction pressure plays a minor role. In addition, the pull-off stress at 50 μm/s is more than twice the atmospheric pressure (~100 kPa), consistent with the findings in previous studies that concluded that van der Waals interactions dominated the attachment at microscale under dry conditions (*7*, *26*).

2) Under water, the behavior is reversed. Attachment is negligible for the substrate with the hole where suction cannot develop, whereas attachment strength exceeds 1 MPa (10 times the atmospheric pressure) for the substrate without hole. The attachment stress increases by two orders of magnitude once the microhole is blocked. Thus, the contact between the lip and the substrate did not contribute to the attachment in the presence of the hole, implying van der Waals contributions to be negligible. Without the hole, hydrostatic liquid pressure contributed to the attachment.

The above experiment, however, does not totally eliminate van der Waals force as a potential contributor, because it does not test its potential emergence in case of substrates without a hole due to high suction pressure between the cup and the substrate. To identify any contribution from van der Waals forces, we need to independently measure the suction pressure inside the cup during retraction in situ. We carry out such pressure measurement by developing a pressure sensor built-in with the substrate.

#### Pressure sensor for the microcup

The sensor consists of a chamber with a diaphragm (Fig. 2, A and B). It is attached to the substrate with an opening so that the pressure inside the cup and that in the sensor chamber are the same. As the stalk is retracted, suction develops, and the sensor diaphragm deforms inward, giving a measure of the pressure. The deflection was quantified from the motion of an attached tongue with respect to a fixed reference (Fig. 2C). A camera was mounted on the side to capture the membrane deflections. ImageJ was used to measure the relative motion between the tongues from the image sequence. Before the suction test in water, the microsensor was degassed to make sure that all the surfaces were fully wet, and no bubble was left inside the sensor cavity.

The sensor was calibrated for pressure-deflection relation (42.1 μm/MPa; section S1 and Materials and Methods). The cup was pressed against the substrate and held for 5 s. The stalk was then retracted with a velocity of 10 μm/s until pull-off. During retraction, force, *F*_0_, on the stalk was recorded by the load cell (fig. S2), while in situ changes in the pressure inside the cup were recorded. At pull-off, the suction pressure was measured as 0.32 MPa. The attachment force can be estimated from the suction pressure as $F_{\text{suction}}=\pi R_{i}^{2}p+\pi (R_{1}^{2}-R_{i}^{2})\frac{p}{2}$, where suction pressure is assumed to vary linearly across the lip width. Figure 2D (also fig. S3) shows the evolution of liquid pressure *p* measured by the sensor, retraction force *F*_0_, and the force *F*_suction_ estimated from the suction pressure, during retraction. Close correspondence between *F*_suction_ and *F*_0_ implies that suction pressure is the primary source of attachment in underwater suction cups, and van der Waals forces did not contribute during retraction. Note that the suction pressure reached more than 0.3 MPa, three times the atmospheric pressure.

### Mechanism of high suction in underwater microcups

We propose a mechanism of the pressure rise and develop a mathematical model for the underwater microcup implementing the proposed mechanism. The predicted pressure and force evolution during retraction at a constant speed, ${\overset{\cdot }{x}}_{0}$, is then compared with the experimental observations. Our working hypothesis is that, during retraction, suction develops inside the cup as well as between the lip and the substrate. Suction pulls the lip toward the substrate, reducing the gap between them and decreasing the flow of water into the cup. This “self-sealing” mechanism leads to a further increase in suction in a feedforward way until the cup detaches.

#### Analytical model of suction cups in liquid

The model (Fig. 2, E and F) has three chambers with elasticity represented by the spring constants *K_i_*, *i* = 1,3. Chamber 1 is axisymmetric and represents the physical suction cup. In the presence of water, herein considered incompressible, its effective stiffness increases to *K*_1_ + *K*_2_, because water constrains its deformation. Chamber 2 accounts for this increased stiffness. Chamber 3 represents the pressure sensor. Chamber 1 has a lip of width *L* = *R*_1_ − *R_i_*. It contacts a substrate with a gap, *d*, between them (Fig. 2G). The contact is frictionless. A vertical spring with stiffness, *K*_0_, represents the stalk of the physical cup in our experiment.

For mimicking a suction microcup experiment, a constant velocity, ${\overset{\cdot }{x}}_{0}$, is applied in the model at the top end of the stalk with force, *F*_0_. Suction pressure, *p*, develops in all the chambers as well as between the lip and the substrate (Fig. 2G). The lip is thus pulled toward the substrate by suction. By our hypothesis, the gap decreases, thus reducing the flow and allowing suction to increase. To model the decrease in gap due to suction, we note that the lip and the substrate are not ideally flat but have asperities. As suction increases, the highest peaks of the asperities first come in contact and deform elastically while smaller peaks come closer and eventually begin to contact. The effective gap, *d*, decreases with increasing *p*, against increasing resistance. Let *F*_lip_ be the net force on the lip (Fig. 2G). We model *d* versus *F*_lip_ relation as $\frac{d(d)}{d(F_{\text{lip}})}≅-d$. This gives

$$d=d_{0}e^{-\frac{F_{\text{lip}}}{F_{*}}}$$ (1)

Here, *d*_0_ is the gap before retraction when *F*_lip_ = 0, and F_*_ is a reference force. Let *Q* be the flow rate into the cup during retraction through the gap *d*. *Q* can be derived using Navier-Stokes equation and the assumption of steady-state flow

$$Q=\alpha \frac{p}{L}d^{3}$$ (2)

Here, α = π*R_i_*/6μ, and μ is the viscosity. During retraction, mass balance gives the governing equation for the evolution of suction inside the cup (section S4)

$$\overset{\cdot }{p}=\beta {\overset{\cdot }{x}}_{0}-\frac{e^{3x_{0}f_{s}}}{\tau }{\mathrm{pe}}^{-\frac{3p}{P_{0}}},p(0)=0$$ (3)

Here, *p*(0) = 0 is the suction at the start of retraction. τ provides a time constant of the suction cup, *P*_0_ is a reference pressure, and β and *f_s_* are constants. Note that τ ∝ μ, *L*. Hence, the higher the viscosity of the fluid or the larger the lip width, the longer is the time needed for the fluid to seep into the suction chamber for a given velocity of retraction.

Next, we compare model predictions with our experimental observations. We carry out a suction cup experiment with ${\overset{\cdot }{x}}_{0}=10\;\mu m/s$, where *F*_0_(*t*) and *p*(*t*) are measured during retraction. Our model predicts a linear relation between ${\overset{\cdot }{F}}_{0}$ and $\overset{\cdot }{p}$ during the entire duration of retraction until pull-off. This linearity is observed experimentally (Fig. 2, D and H). The model predicts linearity between *Y* = *x*_0_ − *p* and $X=\text{ln}(\frac{f(p)}{p})$, whereas the experiment shows a slight deviation from linearity (Fig. 2I). We determine the model parameters *P*_0_, τ, β, and *f_s_* by fitting with the cup experiment (see section S4 and table S1). The evolution of *F*_0_(*t*) predicted by the model also compares well with that observed experimentally at three retraction velocities 5, 10, and 20 μm/s (Fig. 2J). Close correspondence between experimental observation and the model prediction supports the hypothesis that the strength of water glue originates from reducing the gap between the lip and the substrate during retraction; this inhibits the flow of liquid into the cup, resulting in a further increase in pressure in a feedforward way until the cup fails. We explore the failure mechanisms next.

### Detachment mechanisms of suction cup

**Fig. 3.**
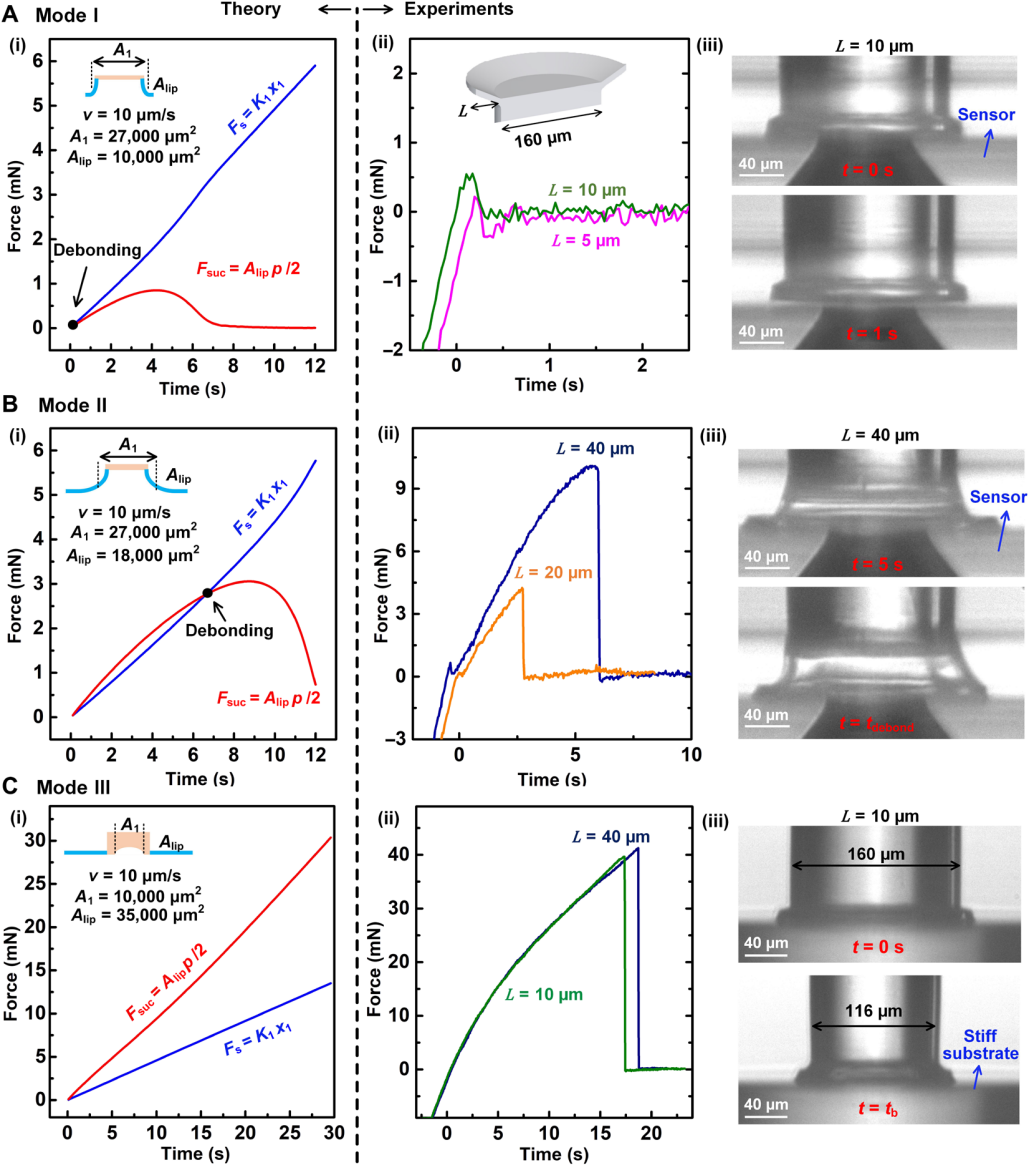
Observation of three detachment modes and comparison with theoretical predictions. (**A**) Mode I: Cup detaches at the start of retraction. (i) Predicted evolution of spring force *F*_s_ and attachment force *F*_suction_ for small cup lip; (ii) experimental data when cup lip *L* is 5 and 10 μm; (iii) in situ images of detachment when *L* = 10 μm. (**B**) Mode II: Cup detaches after suction develops: (i) predicted evolution of spring force *F*_s_ and attachment force *F*_suction_ for large cup lip; (ii) experimental data when cup lip *L* is 20 and 40 μm; (iii) in situ images of detachment when *L* = 40 μm. (**C**) Mode III: Cup detaches by buckling of the lip (out of plane): (i) predicted evolution of spring force *F*_s_ and attachment force *F*_suction_ when tested against a flat substrate; (ii) experimental data when cup lip *L* is 10 and 40 μm against a flat substrate; (iii) in situ images of the detachment when *L* = 10 μm.

One trivial mode (mode I) of detachment occurs when the lip width is small or retraction velocity is low (*18*). The lip does not get pulled in toward the substrate, and suction does not develop. Hence, the detachment force is negligible (Fig. 3A). Here, the rate of increase of pull-off force (spring force in the model) exceeds the rate of suction force at the start of retraction (*t* = 0).

With a larger lip width and velocity of retraction, the lip gets engaged with the substrate, and suction develops. As retraction, *x*_0_, increases, the cup can detach when the suction force on the lip fails to balance the retraction force at the lip-cup junction, i.e., *F*_lip_ = 0 (mode II; Fig. 3B). Here, the rate of increase of suction force on the lip (red line in Fig. 3Bi) exceeds the rate of pull-off force (blue line in Fig. 3Bi) during the early phase of retraction. The lip gets sucked toward the substrate. With further retraction, pull-off force balances the suction force and then exceeds, detaching the cup. Last, even if *F*_lip_ > 0, i.e., suction force on the lip exceeds the pull-off force (red line above the blue line in Fig. 3Ci), the cup may detach abruptly when the lip buckles out of plane because of tangential compressive strain (mode III; Fig. 3C). Buckling leads to the formation of radial channels by the lip, allowing liquid flow from outside, which collapses the suction. We experimentally and computationally test the feasibility of mode III using finite element analysis.

**Fig. 4.**
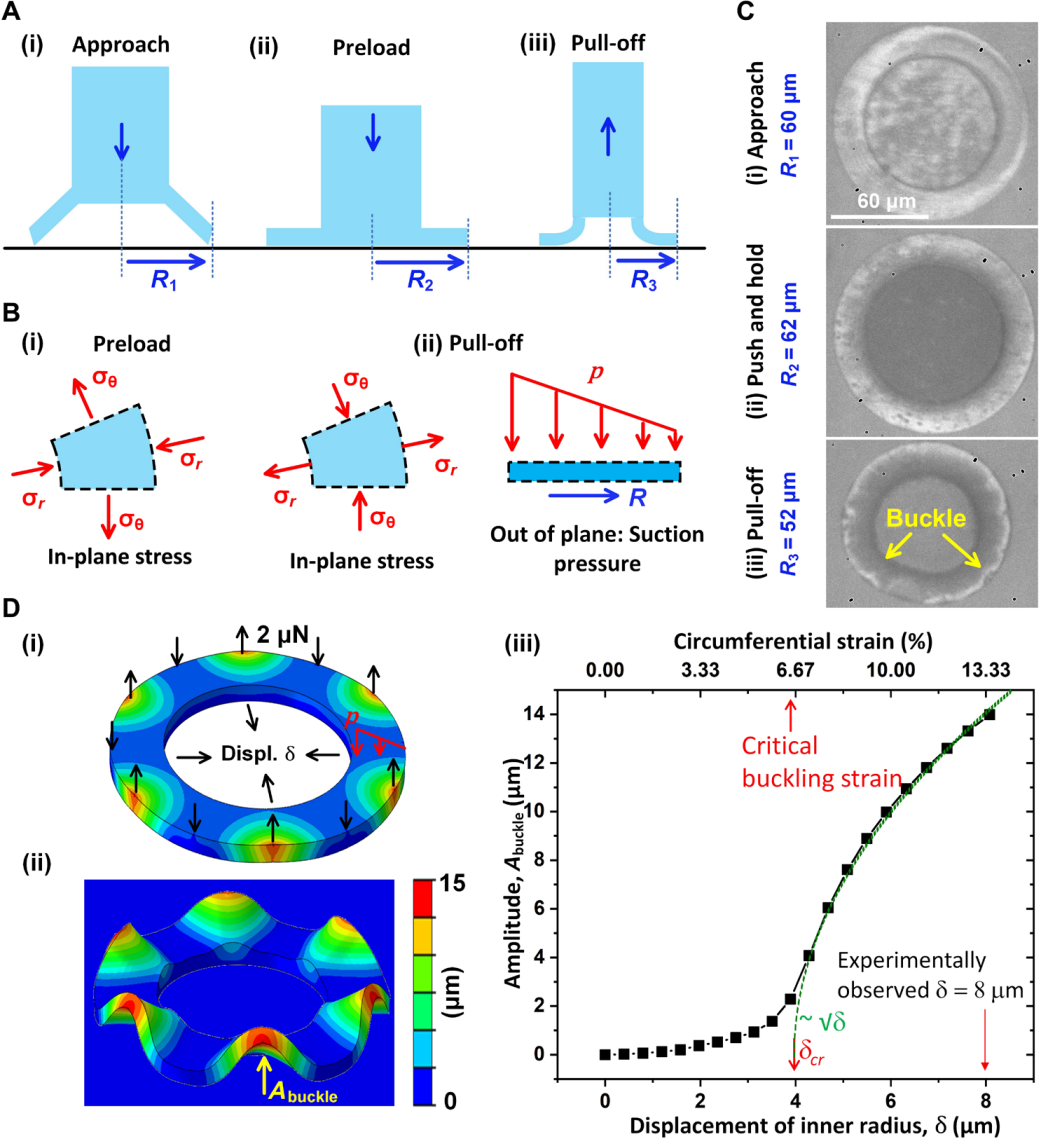
Mechanism of buckling failure during retraction. (**A**) Side-view schematic of the cup: (i) Lip just touches the substrate; (ii) cup is pressed on the substrate with *R*_2_ > *R*_1_; (iii) at pull-off, radius *R*_3_ < *R*_1_. (**B**) Stress states of the lip: (i) After the cup pressed on the substrate, *R*_2_ > *R*_1_, in-plane circumferential stress is tensile but radial stress is compressive. (ii) At pull-off, *R*_3_ < *R*_1_, radial stress is tensile, but circumferential stress is compressive. Strain (compressive) along the outer perimeter of the lip is (*R*_1_ − *R*_3_)/*R*_1_. The lip is under suction pressure *p* toward the substrate. (**C**) Top view of experimental cup during retraction: (i) Cup just touches the substrate with an *R*_1_ of 60 μm; (ii) cup pushed to the substrate with an *R*_1_ of 62 μm; (iii) lip slides inward during retraction. At pull-off, *R*_3_ = 53 μm. (**D**) Finite element analysis of cup-lip buckling: (i) A free plate is subjected to radial inward displacement to identify its lowest mode of buckling. A small point force (2 μN) is applied at the peaks and valleys of the buckled shape (as imperfection) to carry out postbuckling analysis when the plate is subjected to pressure *p*; (ii) postbuckling analysis when the plate is on a frictionless substrate. *A*_buckle_ represents the buckling amplitude; (iii) amplitude of deformation as a function of radial displacement δ at the inner perimeter to identify the onset of buckling at δ*_cr_*.

Mode III proceeds as follows. First, when the cup is pressed against the substrate before retraction, the lip deforms radially outward. Its radius increases and hence is subjected to tangential tensile strain and stress. As illustrated in Fig. 4A, let *R*_1_ be the undeformed radius of the outer rim of the lip. At the pressed configuration, its radius increases to *R*_2_ > *R*_1_. Hence, the tensile strain along the rim is (*R*_2_ − *R*_1_)/*R*_1_. The lip is subjected to tangential tensile stress (Fig. 4Bi). During retraction, the lip slides inward while it is subjected to suction pressure toward the substrate. The rim radius reaches *R*_1_ during retraction while it is still held by the suction. With increasing retraction, the radius decreases with further inward sliding of the lip, and the rim radius decreases to *R*_3_ < *R*_1_. Now, the lip is subjected to a tangential compressive strain of (*R*_1_ − *R*_3_)/*R*_1_. This compressive stress (Fig. 4Bii) would lead the lip to buckle out of the plane. Meanwhile, the lip is subjected to suction (out-of-plane stress) toward the substrate that vanishes at the periphery (Fig. 4Bii) where buckling may initiate. An orthogonal buckling results in a radial channel that allows the fluid to enter the cup. This spontaneous buckling collapses the suction followed by the detachment of the cup.

We test the hypothesis by carrying out a retraction experiment on a microcup against a transparent glass substrate (Fig. 4C). A top-view camera recorded the deformation of the cup and the lip through the glass. The stalk diameter was 80 μm with a cup diameter of 2*R*_1_ = 120 μm. When pushed to the substrate, the diameter of the outer rim of the lip reaches to 2*R*_1_ = 123 μm, with a tensile strain of 2.5%. The stalk was then retracted with a velocity of 10 μm/s when suction pressure holds the lip toward the substrate while allowing it to slide inward. Just before pull-off, the outer diameter of the lip reduces to 2*R*_3_ = 104 μm, giving a compressive strain of 13.3% (Fig. 4Ciii). The image of the lip shows wrinkles around the perimeter, one of which seems to have grown more than the others and might have caused the leakage of water and collapse of the pressure.

The propensity to buckling is confirmed by finite element analysis of an annular plate on a frictionless substrate subjected to vertical pressure mimicking the suction (Fig. 4D; see also section S5). The plate geometry and the material properties of the plate are similar to those of the experimental lip in Fig. 4C: outer radius = 60 μm, inner radius = 40 μm, thickness = 5 μm, elastic modulus = 10 MPa, and Poisson’s ratio = 0.41. A small point load of 2 μN is applied at the buckling locations (peaks) as imperfections to test instability and postbuckling analysis (Fig. 4D, i and ii). The plate is subjected to a radially inward displacement. We find that the plate indeed buckles along the periphery when the outer diameter decreases from 120 to 104 μm, with tangential compressive strain of 13% (Fig. 4Diii), consistent with the experiment.

### Scaling law and design implications

For the design of cups under water, we need to explore how the strength of adhesion scales with cup size. We find in section S5 (Supplementary Materials), to our surprise, that for a given cup design and set of material properties and a prescribed retraction speed, the attachment strength scales inversely with the size of the cup when the cup detaches in mode II, i.e., smaller cups make for stronger attachment under the assumption that water gap *d*_0_ is scaled with size *l* as *d*_0_ ~ *l*^1^, reminiscent of the “smaller is stronger” paradigm for solids (*27*). For a wide variety of aquatic animals using suction cups, attachment strength of their cups scales as (size)^−0.4^ (*28*). In mode III (detachment by buckling), however, the strength is size independent. This is because the buckling strain of the lip is independent of its size scale. This size independence is reminiscent of the critical buckling strain of a Euler beam that is independent of the beam size. It then follows that for a given retraction speed ${\overset{\cdot }{x}}_{0}$, the time *t_b_* to mode III detachment scales as *l*^1^, where *l* represents the size scale of the cup. For a given cup size, *t_b_* decreases with increasing ${\overset{\cdot }{x}}_{0}$ as $t_{b}\sim 1/{\overset{\cdot }{x}}_{0}$. However, time *t_d_* to detachment in mode II scales as the time constant τ of the cup. τ scales with size as τ ~ *l*^3^ if the gap between the lip and the substrate is considered scale independent, i.e., *d*_0_ ~ *l*^0^. This is the case in our experiments where the resolution of three-dimensional printing of the cups or the roughness of the substrate does not change with size. In general, *d*_0_ ~ *l*^1^,when τ, *t_d_* ~ *l*^0^. Neither τ nor *t_d_* depends on ${\overset{\cdot }{x}}_{0}$. We experimentally verified this scaling law in section S6 (fig. S5).

Our scaling analysis thus reveals that, for a given retraction speed, the likelihood of mode III (buckling) detachment increases with cup size (when *d*_0_ ~ *l*^0^), i.e., mode III precedes mode II, because *t_b_* ~ *l*, *t_d_* ~ *l*^3^, and *t_b_* < *t_d_* for large *l*. At a small scale, the likelihood of mode II failure is higher. For a given cup size, detachment mode shifts progressively from mode I to II to III with increasing retraction speed. At low ${\overset{\cdot }{x}}_{0}$, mode I appears immediately upon retraction with negligible attachment strength. At high speeds, *t_b_* is small, and mode III appears suddenly because of buckling instability. At intermediate speeds, *t_d_* < *t_b_*, when mode II precedes mode III. Adhesion strength is highest in mode III, but it is abrupt because of buckling instability. In contrast, mode II is progressive and not abrupt. The cup may remain attached as long as retraction force is balanced by the suction force. Detachment occurs when retraction force exceeds the suction force. This suggests that in nature, mode II might be a preferred mode of detachment, and it is likely to be used for locomotion. Mode III might be reserved for strong attachment for safety and to prevent from being washed away by raging torrents (*15*).

## DISCUSSION

The present study addresses the detailed mechanisms of attachment by deformable microcups. It is clear from the above that water can indeed serve as an adhesive glue between two surfaces, herein a cup and a substrate, under water. From the new insights, we can draw the following conclusions:

1) Strong attachment between two surfaces can be realized in a wet environment by exploiting the water present, not by displacing it. The attachment values found were well above atmospheric pressure and were about one magnitude superior to those under dry conditions.

2) Underwater attachment of deformable microcups is principally different from attachment in air. Whereas the latter is primarily governed by van der Waals interactions, the former relies on a complex interplay between geometry, elasticity, retraction speed, and hydrodynamics.

3) The underwater attachment strength is, under realistic conditions, limited by the elastic properties of the microcup/stalk structure, not by the intrinsic cavitation properties of water. Detachment is preceded by circumferential buckling of the lip of the cup and can be delayed by judicious choice of material parameters.

4) The detachment mechanisms can be conveniently displayed in design maps (fig. S5). These maps and the analysis suggest that miniaturized microcups might be designed with attachment strength limited by water cavitation (above 10 MPa) (*20*).

The mechanism investigated here has promising characteristics for robotics and handling applications, especially under water and in micro dimensions.

## MATERIALS AND METHODS

### Fabrication of cupped microstructures

Cupped microstructures were first printed by two-photon lithography and subsequently replicated from polyurethane (PMC780, Smooth-On, PA, USA). Printed microstructures were then coated by (1*H*,1*H*,2*H*,2*H*perfluorooctyl)-trichlorosilane (AB111444, ABCR, Karlsruhe, Germany) in a vapor deposition method at approximately 5 mbar for 30 min. Then, polydimethylsiloxane (PDMS; Sylgard 184, Dow Corning, Midland, USA) was cast on the cupped microstructures and cured at 75°C for 4 to 5 hours. After demolding, the PDMS structures served as new templates to be replicated by PMC780 polyurethane. Curing of PMC780 was done in an oven at 65°C for at least 12 hours.

### The construction of the pressure sensor

The built-in micro pressure sensor (fig. S1A), designed for monitoring the water pressure inside the microcup, was also printed by a two-photon lithography system (Photonic Professional GT, Nanoscribe, Eggenstein-Leopoldshafen, Germany). Resin IP-S (Nanoscribe, Eggenstein-Leopoldshafen, Germany) was used to create this microsensor. For developing, the microsensor was immersed into propylene glycol monomethyl ether acetate (Sigma-Aldrich, St. Louis, MO, USA) for 30 min. Then, it was rinsed in isopropanol for 1 min and dried by nitrogen, which was repeated four times to completely remove unreacted resin from inside the chamber. Last, the microsensor was postcured by exposing to ultraviolet light (200 mW, 365 nm, OmniCure S1500A, Germany) for 5 min to increase structural stability. The stiffness calibration was done by increasing the pressure difference between the inside and the outside from 0 to 0.2 MPa in steps of 0.05 MPa, while the deflection of the membrane was recorded using an optical microscope (NIKON Eclipse LV100ND). More information can be found in section S1.

### Adhesion measurements

The custom-made adhesion tester consisted of a linear actuator (Q-545.240, PI, Karlsruhe, Germany) to realize precise motion with a resolution of about 6 nm, a load cell (KD45-2N, ME-Messsysteme, Henningsdorf, Germany) to record forces with a resolution of 0.4 mN, and a tubular optic (UltraZoom, Navitar Inc., New York, NY, USA) connected to a camera (DMK 33UX252, ImagingSource, Bremen, Germany) to record videos of the entire tests (fig. S2). At the bottom, the water basin together with the built-in micro pressure sensor were fixed on two goniometers to properly align the sensor to the microcup. Meanwhile, at the top, the cup was glued to the load cell. For the adhesion tests, the cup was moved closer to the sensor with a velocity of 10 μm/s until a preload of 10 mN was reached. After 5 s of contact, the cup was retracted to move upward at a desired velocity (1 to 100 μm/s in the experiments) until a pull-off occurred. Here, the maximum tensile load was defined as the pull-off force. The pull-off stress can be obtained by dividing the force with the projected area of the cup in the original undeformed state.

## Supplementary Materials

### This PDF file includes:

Sections S1 to S6 Figs. S1 to S5 Table S1
