# Supplementary material for: Water as a “glue”: Elasticity-enhanced wet attachment of biomimetic microcup structures
Source: Sci Adv. 2022 Mar 23;8(12):eabm9341. doi: 10.1126/sciadv.abm9341 (PMC8942358; doi:10.1126/sciadv.abm9341)
Supplement: Supplementary file 1 — Sections S1 to S6 Figs. S1 to S5 Table S1 [file sciadv.abm9341_sm.pdf]

Science Advances
AAAS

Supplementary Materials for

Water as a "glue": Elasticity-enhanced wet attachment of biomimetic microcup structures

Yue Wang, Zhengwei Li, Mohamed Elhebeary, René Hensel, Eduard Arzt*, M. Taher A. Saif*

*Corresponding author. Email: eduard.arzt@leibniz-inm.de (E.A.); saif@illinois.edu (M.T.A.S.)

Published 23 March 2022, *Sci. Adv.* **8**, eabm9341 (2022)
DOI: 10.1126/sciadv.abm9341

This PDF file includes:

Sections S1 to S6
Figs. S1 to S5
Table S1

S1. Construction and calibration of the built-in micro pressure sensor

The construction of the pressure sensor

The built-in micro pressure sensor (Figure S1a), designed for monitoring the water pressure inside the micro-cup, was printed by a two-photon lithography system (Photonic Professional GT, Nanoscribe, Eggenstein-Leopoldshafen, Germany). Resin IP-S (Nanoscribe, Eggenstein-Leopoldshafen, Germany) was used to create this micro sensor. For developing, the micro sensor was immersed into propylene glycol monomethyl ether acetate (PGMEA, Sigma-Aldrich, St. Louis, MO, USA) for 30 min. Then, it was rinsed in isopropanol for 1min and dried by nitrogen which was repeated 4 times to completely remove unreacted resin from inside the chamber. Finally, the micro sensor was post-cured by exposing to UV light (200 mW, 365 nm, OmniCure S1500A, Germany) for 5 min to increase structural stability.

The deformable membrane inside the sensor deflects due to difference in pressure inside and outside the sensor chamber. It is 5 μm thick and 300 μm in diameter. An extension (tongue) with a diameter of 50 μm fixed at the center of the membrane moves with the membrane deflection. It gives a measure of deflection with respect to a reference beam (fixed tongue) on the side wall (Figure S1a-ii). Except the deformable membrane (5 μm thick), other walls building the cavity of the sensor were relatively stiff with a thickness of 50 μm. The sensor has a 80 μm opening at the top (Figure S1a-iii) for continuity of fluid between the cup and the sensor.

The stiffness calibration of the micro sensor

To calibrate the sensor, the stiffness of the membrane was determined. Therefore, the opening of the sensor was sealed using 10 wt% polyvinyl alcohol powder dissolved in water. A small droplet was carefully placed at the opening of the sensor and, subsequently, dried for 3 h at room temperature (Figure S1b-i). The sealed sensor was then placed in the pressure chamber. The pressure difference between inside and outside was increased from 0 to 0.2 MPa in steps of 0.05 MPa. Meanwhile, the deflection of the membrane was recorded using an optical microscope (NIKON Eclipse LV100ND). The calibration process was repeated twice. Figure S1b-iii shows the linear increase of the membrane deflection with increasing pressure, providing a stiffness of 42.1 μm/MPa.

S2. Fabrication of the cupped microstructure and test setup

Fabrication of cupped microstructures

Cupped microstructures were first printed by two-photon lithography and, subsequently, replicated from polyurethane (PMC780, Smooth-On, PA, USA). The procedure of the two-photon lithography was the same as described in section S1. Printed microstructures were coated by (1H,1H,2H,2Hperfluorooctyl)-trichlorosilane (AB111444, ABCR, Karlsruhe, Germany) in a vapor deposition method at approx. 5 mbar for 30 min. Then, polydimethylsiloxane (PDMS, Sylgard 184, Dow Corning, Midland, USA) was cast on the cupped microstructures and cured at 75 °C for 4 to 5 h. After demolding, the PDMS structures served as new templates to be replicated by PMC780 polyurethane. Curing of PMC780 was done in an oven at 65 °C for at least 12 h.

Water degassing process

Prior to all tests under water with the built-in micro pressure sensor, the trapped air inside the sensor chamber had to be removed. Degassing at 50 mbar for 30 min was often not sufficient to remove air bubbles through the small opening of the sensor. Therefore, an alternative procedure was employed: First, 2 ml of a water-ethanol mixture (volume ratio 1:2) was added to the already immersed micro sensor, followed by degassing at 50 mbar for 10 min at room temperature. The lower surface free energy and higher wettability of the ethanol-water mixture (compared to pure water) facilitated bubbles to burst out. After degassing, the micro-sensor, which was inundated in a water and ethanol mixture, was diluted in 500 ml deionized water for three times to remove ethanol. The micro sensor was then immersed in a water basin (Figure S2a) with about 5 ml deionized water ready for the adhesion tests.

Adhesion measurements

As shown in Figure S2a, the custom-made adhesion tester consisted of a linear actuator (Q-545.240, PI, Karlsruhe, Germany) to realize precise motion with a resolution of about 6 nm, a load cell (KD45-2N, ME-Messsysteme, Henningsdorf, Germany) to record forces with a resolution of 0.4 mN, and a tubular optic (UltraZoom, Navitar Inc., New York, NY, USA) connected with a camera (DMK 33UX252, ImagingSource, Bremen, Germany) to record videos of the entire tests. At the bottom, the water basin together with the built-in micro pressure sensor were fixed on two goniometers to properly align the sensor to the micro cup. Meanwhile, at the top, the cup was glued to the load cell.

Before the tests, the center of the cup was aligned with the center of the opening of the micro sensor. This was achieved by two step motors (VT-80 linear stages, PI, Karlsruhe, Germany) and visualization with a camera. For the adhesion tests, the cup was approached to the sensor with a velocity of 10 μm/s until a preload of 10 mN was reached. After 5 seconds of contact, the cup was retracted to move upward at a desire velocity (1~100 μm/s in the experiments) until a pull-off occurred. Here the maximum tensile load was defined as the pull-off force. The pull-off stress can be obtained by dividing the force with the projected area of the cup in the original undeformed state.

S3. Recorded membrane deflections

Figure S3 gives the *in-situ* images with the membrane position at different retraction times as well as the deflection of force sensing membrane and the corresponding liquid pressures at different time points of retraction at the retraction velocity of $\dot{x}_0 = 10\ \mu\mathrm{m/s}$.

S4. Analytical model of underwater suction cup and its parameter estimation

We develop a simple mathematical model of the cup with the sensor (Figure 2f). The model has three chambers, 1-3, with spring constants K_1, K_2, and K_3. Chamber 1, representing the cup, is axi-symmetric with cross sectional area A_1. It has a lip with width L that comes in contact with a substrate. The contact is frictionless. A vertical spring with stiffness, K_0, represents the stalk. Let x_0 be the applied prescribed retraction at the top end of the stalk, and F_0 is the corresponding measured force. Let x_1 be the corresponding displacement at the base of the stalk. x_1 accounts for the elastic deformation of the cup, as well as inward sliding and deformation of the lip. We employ a simple model with $\frac{dF_0}{dx_1} = K_1$, the spring constant of Chamber 1 (Figure 2e and 2f). Note that there is no fluid involved in defining K_1, and hence there is no pressure change in the chamber due to x_1.

The effective stiffness of the cup increases in the presence of liquid, herein considered incompressible. If liquid is not allowed to seep into the cup through the space between the lip and the substrate, then the deformation of the cup must satisfy volume conservation. Thus, during retraction, the cup cannot deform as freely, and its effective stiffness increases. Chamber 2 in the model accounts for this increased stiffness. Chambers 1 and 2 are connected, and hence

they share the same suction pressure, p. When the stalk is retracted, volume conservation requires $x_1A_1 = x_2A_2$, where A_2 and x_2 are the cross-sectional area and the deformation of chamber 2. Force balance requires $pA_2 = K_2x_2$. Now, force, F_0, required to move the stalk by x_0 is $F_0 = K_0(x_0 - x_1)$. $x_1$is obtained from force balance $K_0(x_0 - x_1) = K_1x_1 + A_1p$. Then F_0 is given by

$$F_0 = K_1x_1 + A_1p = \frac{K_0}{K_0+K_1}(A_1p + K_1x_0). \tag{S1}$$

Chamber 3 represents the pressure sensor. It has a cross sectional area of A_3 and spring constant, K_3. It is connected to chambers 1 and 2, and hence shares the same suction pressure, p with $pA_3 = K_3x_3$. If liquid is not allowed to seep in from outside during retraction, then mass conservation requires $A_1x_1 = A_2x_2 + A_3x_3$. Allowing the flow, let Q be the flow per unit time into the suction cup as the stalk is retracted. Then mass balance requires:

$$A_1\dot{x}_1 = A_2\dot{x}_2 + A_3\dot{x}_3 + Q. \tag{S2}$$

For an axisymmetric case, Q can be derived using Navier-Stokes equation and the assumption of steady state condition. Q is given by

$$Q = \frac{-2\pi(p_i - p_{out})d^3}{12\mu\ln\left(\frac{R_i}{R_o}\right)} \approx \frac{\pi}{6\mu}(p_{out} - p_i)d^3\frac{R_i}{L} = \frac{\pi}{6\mu}\frac{R_i}{L}pd^3 = \alpha\frac{p}{L}d^3. \tag{S3}$$

Here p_{out} and p_{in} are the water pressures outside and inside the suction cup, $p = p_{out} - p_i$ is the suction pressure in the cup, $\alpha = \frac{\pi R_i}{6\mu}$, μ is the viscosity and R_i is the inner radius of the lip. Eq.(S1)-(S3) with force balance conditions, $pA_3 = K_3x_3$ and $pA_2 = K_2x_2$, give the evolution equation for pressure as the stalk is retracted with a prescribed velocity, $\dot{x}_0$:

$$\left(\frac{A_1^2}{K_0+K_1} + \frac{A_2^2}{K_2} + \frac{A_3^2}{K_3}\right)\dot{p} = \frac{K_0}{K_0+K_1}A_1\dot{x}_0 - \alpha\frac{p}{L}d^3, \quad p(0) = 0. \tag{S4}$$

We note that suction pressure develops between the lip and the substrate over the width, L, of the lip (Figure 2g). Suction reaches the value of p at the inner periphery of the lip. Suction vanishes at the outer periphery of the lip. The lip is thus pulled towards the substrate by the suction. Both the lip and the substrate have asperities. As suction increases, the highest peaks first come in contact and deform elastically while smaller peaks come closer and eventually begin to contact. The effective gap, d, decreases with increasing p, but with increasing resistance. As shown in Figure 2g, the net force on the lip is given by $F_{lip} = \frac{A_{lip}p}{2} - F_s$. F_{lip} is balanced by the resistance of the substrate. Here $A_{lip} = 2\pi\left(R_i + \frac{L}{2}\right)L$ is the area of the lip, and F_s is the force of the spring from chamber 1. Higher

the net force, F_{lip}, smaller is d and higher is the resistance to further decrease in d. We thus model d versus F_{lip} relation as: $\frac{d(d)}{d(F_{lip})} \cong -d$. This gives

$$d = d_0 e^{-\frac{F_{lip}}{F_*}}. \tag{S5}$$

Here d_0 is the gap when $F_{lip} = 0$, and F_* is a reference F_{lip} at which $\frac{d}{d_0} = e^{-1} \approx \frac{1}{3}$. We expect that $|x_1| \gg |\Delta|$, where $\Delta = d - d_0$ is the change in d. This gives $F_s = K_1(x_1 - \Delta) \approx K_1 x_1$. Thus,

$$F_{lip} = \frac{A_{lip}p}{2} - K_1 x_1 = \left(\frac{A_{lip}}{2} + \gamma A_1\right) p - \gamma K_0 x_0. \tag{S6}$$

Here $\gamma = \frac{K_1}{K_1+K_0}$. Let $F_* = \left(\frac{A_{lip}}{2} + \gamma A_1\right) P_0$ where P_0 be a reference pressure. Then, from Eqs. (S5) and (S6),

$$d = d_0 e^{-\frac{p}{P_0}} e^{f_s}\ ,\ f_s = \frac{\gamma K_0 x_0}{F_*}. \tag{S7}$$

Mass balance (Eq. S4) then gives:

$$\dot{p} = \beta \dot{x}_0 - \frac{e^{3f_s}}{\tau} p e^{-\frac{3p}{P_0}}, p(0) = 0. \tag{S8}$$

$$\text{Here, } \tau = \frac{L\left(\frac{A_1^2}{K_0+K_1} + \frac{A_2^2}{K_2} + \frac{A_3^2}{K_3}\right)}{\alpha d_0^3},\ \beta = \frac{\frac{K_0 A_1}{K_0+K_1}}{\left(\frac{A_1^2}{K_0+K_1} + \frac{A_2^2}{K_2} + \frac{A_3^2}{K_3}\right)} \tag{S9}$$

τ serves as a time constant of the suction cup. Note that, since $\alpha = \frac{\pi R_i}{6\mu}$, hence the time constant, $\tau \propto \mu, L$, i.e., higher the viscosity of the fluid or longer the lip width, longer is the time needed for the fluid to seep into the suction chamber for a given velocity of retraction.

<u>Remarks:</u> Eq. (S8) represents a simple mathematical model of suction cups in liquid. It predicts the time evolution of pressure in the cup during retraction. The parameters that characterize the cup are: time constant τ, reference pressure P_0, geometric and stiffness parameter β, chamber area A_1 and stiffness K_1. Simple mathematical models are often used to gain physical insight on complicated physical systems. Examples include single degree of freedom (SDOF) model representing dynamical systems, Maxwell and Kelvin-Voigt models representing viscoelastic systems. The validity of such simple models is verified by comparing the trends of the system behavior predicted by the model with those observed experimentally in the physical system. We follow a similar strategy below. Experimentally, the measured quantities are, $x_0(t)$, $F_0(t)$, and chamber pressure, $p(t)$, recorded by the pressure sensor. In the following,

we study the model using the experimental cup tested in Figure 2 and Figure S2, with dimensions as follows: stalk radius, $R_i = 80\ \mu m$, stalk length, $L_{stalk} = 250\ \mu m$, lip radius, $R_1 = 120\ \mu m$, lip thickness, $T = 10\ \mu m$, elastic modulus of cup material, $E \approx 10\ MPa$, Poisson's ratio, $\upsilon = .41$. We will estimate the parameters of the model corresponding to the physical cup, as well as compare model predictions with experimental observations. We determine the model parameters, P_0, β, A_1, K_1 and τ for the cup firstly with $\dot{x}_0 = 10\ \mathrm{\mu m/s}$.

Determination of A_1 and K_1:

Eq. (S1) gives the time derivative of the force, $\dot{F}_0$, as the stalk is retracted:

$$\dot{F}_0 = \frac{K_0}{K_0 + K_1} A_1 \dot{p} + \frac{K_0 K_1}{K_0 + K_1} \dot{x}_0 \qquad \text{(S10)}$$

where K_0 is the stiffness of the stalk given by $K_0 = \frac{EA_{stalk}}{L_{stalk}} = 0.8 \frac{\mathrm{mN}}{\mathrm{\mu m}}$. Here, E is the elastic modulus, A_{stalk} the cross-sectional area of the stalk, and L_{stalk} the stalk length. Using the data shown in Figure 2d and Figure S3b for the retraction velocity, $\dot{x}_0 = 10\ \mathrm{\mu m/s}$, we plot $\dot{F}_0$ versus $\dot{p}$ for the duration of 6 s (Figures 2h and S4a). The model (Eq. (S10)) predicts a linear relation between them. Experiment verifies the linearity. The slope of the linear fit gives $K_0 A_1 (K_0 + K_1)$ and the intercept gives $\frac{K_0 K_1}{K_0 + K_1}$. Thus, $A_1 = 31361\ \mathrm{\mu m^2}$ and $K_1 = 0.0579\ \mathrm{mN/\mu m}$.

Determination of β:

Eq. (S8) gives $\dot{p} = \beta \dot{x}_0$ at $t = 0$, since $p(0) = 0$. Then, Eq. (S10) can be used to evaluate β from experimentally measured $\dot{F}_0(t = 0)$. Figure S4b shows $\dot{F}_0(0)$ for $\dot{x}_0 = 10\ \mathrm{\mu m/s}$. Since, we already have A_1 and K_1,

$$\beta = \frac{\dot{p}}{\dot{x}_0} = \left(\dot{F}_0 - \frac{K_0 K_1}{K_0 + K_1} \dot{x}_0 \right) \Bigg/ \left(\frac{\frac{K_0}{K_0 + K_1} A_1}{\dot{x}_0} \right) = 6.46 \cdot 10^{-6}\ \mathrm{mN/\mu m^3}$$

Determination of time constant, τ, and reference pressure, P_0

We rewrite Eq. (S8) by taking a log on both sides and defining $f(p) = \beta \dot{x}_0 - \dot{p}$ (Figure S4c) as

$$P_0 f_s - p = \frac{P_0}{3} \ln(\tau) + \frac{P_0}{3} \ln\left(\frac{f(p)}{p} \right) \qquad \text{(S11)}$$

From Eq. (S7), $f_s = \gamma K_0 x_0 / F_*$ and $F_* = \left(\frac{A_{lip}}{2} + \gamma A_1 \right) P_0$, we have

$$P_0 f_s = \frac{\gamma K_0 x_0}{\frac{A_{lip}}{2} + \gamma A_1} \tag{S12}$$

Let $Y = \frac{\gamma K_0 x_0}{\frac{A_{lip}}{2} + \gamma A_1} - p$ and $X = \ln\left(\frac{f(p)}{p}\right)$. Then, Eq. (S11) becomes:

$$Y = \frac{P_0}{3}\ln\tau + \frac{P_0}{3}X \tag{S13}$$

The model (Eq. (S13)) predicts a linear relation between $X(t)$ and $Y(t)$ for a given P_0. Experimentally, we have the values of $x_0 = \dot{x}_0 t$, $\gamma = \frac{K_1}{K_1 + K_0}$, A_1, and $A_{lip} = 2\pi\left(R_i + \frac{L}{2}\right)L$ for the cup in Figure 2. We also measured suction pressure, $p(t)$, using the pressure sensor for a duration of about 6 s until the cup detaches. We thus plot Y versus X using the experimental values (Figure 2i, Figure S4d). In contrast to the model prediction, they deviate slightly from linearly. In order to obtain τ and P_0, we fit a straight line through the test data. The slope and intercept are $\frac{P_0}{3} = 1.7 \cdot 10^{-4}$ and $\frac{P_0}{3}\ln\tau = 3.48 \cdot 10^{-4}$. These give $\tau = 7.75$ s and $P_0 = 5.1 \cdot 10^{-4}\ \mathrm{mN/\mu m^2} = 0.51$ MPa.

The above model parameters are evaluated for retraction velocity of $\dot{x}_0 = 10\ \mu\mathrm{m/s}$. We also evaluated the parameter values for $\dot{x}_0 = 1\ \mu\mathrm{m/s}$ and $\dot{x}_0 = 100\ \mu\mathrm{m/s}$, varying over two orders of magnitude (Table S1). Note that, except for the time constant τ, other parameters remain nearly constant for different speeds. τ is much shorter for higher speeds. The speed dependence of τ may originate from viscoelasticity of cup material, polyurethanes. The model considers the material as linear elastic. In order to correct this discrepancy, we apply a correction factor for the time constant, as τ_v (velocity dependent time constant) as

$$\tau_v = \tau\frac{1}{(1+\dot{x}_0)^c} = \frac{L\left(\frac{A_1^2}{K_0 + K_1} + \frac{A_2^2}{K_2} + \frac{A_3^2}{K_3}\right)}{\alpha d_0^3}\frac{1}{(1+\dot{x}_0)^c} \tag{S14}$$

Taking log on both sides, $\ln(\tau_v) = \ln(\tau) - c\ln(1+\dot{x}_0)$, and linearly fitting the data in the log-log plot (Figure S4e), we can get: $c = 0.92$. Note that $\dot{x}_0$ in Eq. (S14) can be considered as normalized by 1 μm/s, i.e., $\dot{x}_0$ is non-dimensional. Figure S4e further shows that the variation in the rest of the parameters (P_0, β, A_1, K_1) is small even when the retraction velocity changes by two orders of magnitude. The similarity between the predicted and experimental trends in cup response discussed above suggests that the model represents the suction cups in liquids. To further test the model, we conduct retraction experiment with $\dot{x}_0 = 5$, and $20\ \mu\mathrm{m/s}$ and measure the corresponding force of retraction until detachment. We also apply the model to predict the force-time history using the parameters. The model predictions almost coincide with experimental observation (Figure 2j, Figure S4f), except near detachment or failure, which we discuss next.

S5. Failure of the cup by buckling: finite element analysis

In order to test whether the lip of the cup can buckle during retraction, we simulate the deformation of the lip using finite element analysis (Figure 4d). The lip geometry and material properties are similar to those in the experiment (Fig. 4c, outer lip radius = 60 μm, stalk radius = 40 μm, thickness = 5 μm, elastic modulus = 10 MPa, Poisson's ratio = 0.41). However, the lip is treated as an independent plate isolated from the stalk. The analysis consists of two stages: (1) Eigen analysis of the lip to determine the lowest buckled mode shape. The inner perimeter of the lip is constrained from out of plane displacement, but it is subjected to an inward radial displacement. The rest of the lip is free. At a critical inward displacement, the lip buckles. This gives the mode shape of the lip at buckling (Figure 4d-i). (2) In order to simulate the experimental condition of retraction, we now model the lip on a substrate, where lip-substrate contact is frictionless. The lip can deform out of plane away from the substrate. In order to mimic suction as in case of the experiments, an out-of-plane pressure is applied on the plate that decreases radially outward. The pressure is highest (1 MPa) at the inner perimeter, and it vanishes at the outer perimeter (Fig. 4d-i). The pressure pushes the lip towards the substrate. In order to test potential buckling, we apply small point loads (2 μN) at discrete locations along the outer perimeter. These locations are the peaks (2 μN pointing upward) and valleys (2 μN pointing downward) of the free buckled plate (Fig. 4d-i). These small loads induce "imperfections" in the plate, and they allow to carry out post buckling analysis. The inner perimeter of the lip is restricted to in-plane displacement only. It is subjected to radially inward displacement, mimicking radial motion during retraction. If buckling is energetically favorable under these constraints, then we expect a non-linear relation between the radial displacement and out of plane deformation along the point loads. The amplitude, A_{buckle}, of the out-of-plane deformation of the lip at the locations of 2 μN load increases with the radial motion of the outer perimeter. A_{buckle} is expected to increase rapidly near a critical δ_{cr} (Figure 4d-iii). Without any imperfection, A_{buckle} is expected to increase as $\sqrt{\delta}$ when $\delta > \delta_{cr}$. We thus fit a $\sqrt{\delta}$ curve to the simulation results of δ vs A_{buckle}. The curve meets the x-axis at $\delta_{cr} \approx 4$ μm when the circumferential strain is about 6.7%. We consider this strain as the critical buckling strain for the underwater cup. This strain cannot be detected experimentally. Once buckling instability initiates, the amplitude increases rapidly with retraction. The experimental image prior to detachment shows (Figure 4c) that the outer diameter of the lip is 52 μm, implying a strain of 13.5%. Simulation also shows buckling amplitude of about 14 μm at a strain of about 13.3%.

S6. Effect of scaling on attachment strength

We derive the scaling law from the analytical model described above. We consider two cases for scaling: $d_0 \sim (size)^1$, and $d_0 \sim (size)^0$. The former represents aquatic animals in natural biological contacts, and the latter reflects our experimental setup where the resolution of 3D printing of the cups or the roughness of the substrate do not change with cup size. In our scaling analysis, we assume $d = d_0$ in Eq. S5, i.e., the gap between the lip and the substrate does not change with suction. Let l represent the size scale of the cup.

Consider a cup without a built-in sensor, i.e., $K_3 \to \infty$. Then, during retraction of the stalk with constant velocity, $\dot{x}_0$, the suction pressure evolves as (Eq. S4, S8-9)

$$p(t) = \tau\beta\dot{x}_0\left(1 - e^{-\frac{t}{\tau}}\right),\ \tau = \frac{A^2\left(\frac{1}{K_0+K_1}+\frac{1}{K_2}\right)L}{\alpha d_0^3},\ \beta = \frac{\frac{K_0}{K_0+K_1}}{\left[A\left(\frac{1}{K_0+K_1}+\frac{1}{K_2}\right)\right]},\ \alpha = \pi R_i/6\mu \qquad \text{(S15)}$$

From Eq. S1, corresponding force of retraction increases as

$$F(t) = \frac{K_0}{K_0+K_1}\left(p(t)A + K_1 x_0(t)\right) \qquad \text{(S16)}$$

In arriving at Eq. S15 and S16, we used $A = A_1 = A_2$ in Eqs. S1, S4, S8-9 without los of generality. For our experimental cups, $A \approx A_{stalk}$ is the area of the stalk cross section, and R_i is the stalk radius ($A = \pi R_i^2$). For linear elastic solids, stiffness $K \sim l^1$. Hence, $\beta \sim l^{-1}$. If we consider $d_0 \sim l^1$ then $\tau \sim l^0$, i.e., independent of cup size. If d_0 does not change with cup size, i.e., $d_0 \sim l^0$, then $\tau \sim l^3$.

To determine the scale dependence of strength, we note that the cup may detach in three independent modes during retraction. Mode I: The lip does not get pulled-in towards the substrate and suction does not develop. Here, detachment occurs as soon as the stalk is retracted. This occurs if the *rate* of increase of retraction force exceeds the *rate* of increase of suction force at time, $t = 0$. This mode is avoided by increasing the rate of increase of suction force for a given rate of retraction, $\dot{x}_0$, i.e., by increasing the lip width such that $\frac{A_{lip}}{2A} > K_1/K_2$. Once the lip is engaged, then the cup may detach either by detaching when the retraction force exceeds the suction force (Mode II), or by buckling the lip out of plane (Mode III). Mode II detachment occurs when $F_{lip} = 0$ (Eq S6). This gives

$$1 - e^{-\frac{t_d}{\tau}} = a t_d \qquad \text{(S17)}$$

Here, t_d is the time to detachment in Mode II. t_d can be solved from Eq. S17 for a given $a = \frac{\alpha d_0^3 K_1}{AL\left(\frac{A_{lip}}{2}+\gamma A\right)}$. $a \sim l^{-3}$ when $d_0 \sim l^0$; and $a \sim l^0$ when $d_0 \sim l^1$. From Eq. S17, $\frac{t_d}{\tau}, at_d \sim l^0$. Thus, $t_d \sim l^3$ when $d_0 \sim l^0$, and $t_d \sim l^0$ when $d_0 \sim l^1$. At the impending detachment (Mode II, $F_{lip} = \frac{A_{lip}p}{2} - K_1 x_1 = 0$), attachment force, F_d is given by Eqs. S1, S15 and S17:

$$F_d = \left(A + \frac{A_{lip}}{2}\right)p = \left(A + \frac{A_{lip}}{2}\right)\dot{x}_0 \tau \beta \left(1 - e^{-\frac{t_d}{\tau}}\right) \qquad \text{(S18)}$$

Then, attachment strength, $\sigma_{max}^d = \frac{F_d}{A+\frac{A_{lip}}{2}} \sim l^3 l^{-1} l^0 \sim l^2$ when $d_0 \sim l^0$, i.e., strength decreases with size, as expected, since a fixed d_0 would appear large as cup size becomes very small. The cup will offer little adhesion due to fluid entrée to the cup through the relatively large gap, d_0. But $\sigma_{max} \sim l^0 l^{-1} l^0 \sim l^{-1}$ when $d_0 \sim l^1$, i.e., smaller cups would become stronger. If d decreases with increasing suction, then the strength is espected to be even higher. Softer the lip, higher is its deformability, and more conformal it will be with the substrate with suction. This will decrease the gap further and increase the cup strength.

At buckling (Mode III), Eq. S1 gives the detachment force

$$F_b = \frac{K_0}{K_0+K_1}\left(p(t_b)A + K_1 x_{0b}\right) \qquad \text{(S19)}$$

Here x_{0b} is the displacement of the stalk at the time, t_b, when buckling initiates. If ε_b is the circumferential compressive strain of the lip (outer rim) at buckling, then $x_{0b} = \dot{x}_0 t_b \approx \varepsilon_b(R_i + L) + \varepsilon_{stalk} L_{stalk}$. Here, ε_{stalk} is the elastic strain of the stalk of length L_{stalk}, ε_b is the buckling strain (circumferential) at the lip periphery, given by, $\varepsilon_b = \Delta(R_i + L)/(R_i + L)$. The approximation above is because a fraction of $\Delta(R_i + L)$ contributes to x_{0b}. Since both ε_{stalk} and ε_b are the elastic strains induced by F_b, ε_{stalk} and ε_b are proportional to each other. Since ε_b is the threshold strain for buckling, it is scale independent, just as buckling strain of Euler column is independent of its size. Then, ε_{stalk} at buckling is also size independent. Thus, $x_{0b} \sim l^1$ and $t_b = \frac{x_{0b}}{\dot{x}_0} \sim l^1$ for a given $\dot{x}_0$. It follows that larger the cup size, the longer it takes to initiate buckling.

From Eqs. S15 and S19, detachment force at buckling, F_b, is

$$F_b = A\dot{x}_0 \tau \beta \frac{K_0}{K_0+K_1}\left(1 - e^{-\frac{t_b}{\tau}}\right) + \frac{K_0 K_1}{K_0+K_1} x_{0b} \qquad \text{(S20)}$$

Consider the case $d_0 \sim l^0$. Here, $\tau \sim l^3$ and $\frac{t_b}{\tau} \sim l^{-2}$. For small cups (small l), $t_b/\tau \to \infty$, and $\left(1 - e^{-\frac{t_b}{\tau}}\right) \approx 1$. For large cups (large l), $t_b/\tau \to 0$ and $\left(1 - e^{-\frac{t_b}{\tau}}\right) \approx t_b/\tau$. From Eq. S20, attachment strength scales as $\sigma_{max}^b = \frac{F_b}{cup\ area} \sim l^2 + l^0 \sim l^0$ for small cups, and $\sigma_{max}^b \sim l^0 + l^0$ for large cups. Now, consider the case of $d_0 \sim l^1$. Here, $\tau \sim l^0$ and $\frac{t_b}{\tau} \sim l^1$.

Then, for small cups, $t_b/\tau \to 0$, and $\left(1 - e^{-\frac{t_b}{\tau}}\right) \approx t_b/\tau$. For large cups, $t_b/\tau \to \infty$ and $\left(1 - e^{-\frac{t_b}{\tau}}\right) \approx 1$. From Eq. S20, $\sigma_{max}^b \sim l^0 + l^0 \sim l^0$ for small cups, and $\sim l^{-1} + l^0 \sim l^0$ for large cups. This implies that attachment strength in buckling mode is independent of cup size for a prescribed $\dot{x}_0$.

Cups can be designed with specific desired failure mode. For example, one can design a cup that reaches failure by detaching in Mode II when $t_d = \tau$. Eqs. S15 and S17 then give the necessary geometric condition:

$$\gamma + \frac{3K_1}{K_2} = \frac{A_{lip}}{A} > \frac{2K_1}{K_2} \qquad \text{(S21)}$$

Here, $e^{-1} \approx 1/3$ is used. The latter inequality in Eq. S21 prevents failure by Mode I. To ensure that buckling does not occur before Mode II debonding, we require $t_b > \tau$, which gives

$$\frac{x_{0b}}{\dot{x}_0} = (\varepsilon_b(R_i + L) + \varepsilon_{stalk}L_{stalk})/\dot{x}_0 > 6\pi\mu L(\frac{R_i^3}{d_0^3})(K_0 + K_1 + K_2)/(K_2(K_0 + K_1)) \qquad \text{(S22)}$$

For a given retraction speed of $\dot{x}_0$, the above condition can be met by reducing lip width L, and R_i, and by tuning the stiffnesses, K_i.

Recall that time to buckling $t_b \sim l$, and time to de-bond in mode II, $t_d \sim l^3$ (when $d_0 \sim l^0$, our experimental case). Thus, as the cup size increases, buckling mode is expected to precede the debonding mode. When $d_0 \sim l^1$, then $t_d \sim l^0$ while t_b remains as $t_b \sim l$. Hence, debonding failure by Mode II is likely to occur with increasing size. For a given cup size, $t_b$increases with decreasing retraction speed, $\dot{x}_0$ as $t_b \sim 1/\dot{x}_0$, whereas t_d is independent of $\dot{x}_0$. Thus, for a given cup, the detachment mode changes with increasing $\dot{x}_0$ from Mode II to Mode III.

Experimental verification of the scaling law:

We fabricated 6 cups (design shown in Fig 1b) with R_i varying from 40 to 320 µm, lip thickness $T = 0.1R_i$ (i.e., $q = 0.1$), lip radius $R_1 = 1.2\, R_i$. Due to fabrication limitation, the height of the stalk, L_{stalk}, could not be scaled as Ri, and was limited to 250 µm as follows: (R_i, L_{stalk}) = $(40\ \mu m, 125\ \mu m)$, $(80, 250)$, $(160, 250)$, $(240, 250)$, $(280, 250)$, $(320, 250)$. The cups were fabricated with polyurethane with elastic modulus of 10 MPa and 4 MPa. All cups were subjected to a high retraction speed of $\dot{x}_0 = 50\ \mu m/s$. They all detached in Mode III (buckling of the lips) and their attachment strength remained nearly constant with change in size by nearly an order of magnitude, but strength increased with increase in elastic modulus, E, of cups (Fig. S5).

We derive three insights from these observations in relation to our model and scaling laws.

(1) Our model (Eqs. S20) predicts that the pull-off strength is size independent in Mode III, $\sigma_{max}^b \sim {R_i}^0$. This is observed in the experiments when R_i increased from 40 to 80 µm.

(2) We observed that strength remained almost constant when R_i increased from 80 to 320 µm while the stalk length, L_{stalk}, remained fixed. To explain this, we suspect that due to high speed of retraction, in all cases, the time to Mode III detachment was much shorter than the time constant of the cups, i.e., $t_d \ll \tau$. Indeed, experimentally, we find t_d varying in 5-7 s as R_i increased from 80 to 320 µm, while $L_{stalk} = 250\ \mu m$. Eq. S15 predicts $\tau = 560\ s$ to 36000 s for R_i (80, 320 µm) with an estimated $d_0 = 10\ nm$. This implies that the exponential term on the right side of Eq. S20 vanishes, and the second term, $\frac{K_0 K_1}{K_0+K_1} x_{0b}$ contributes mostly to the attachment force, F_b. Increasing R_i with constant stalk length increases K_0 as $K_0 \sim {R_i}^2$. Since $K_1 \sim {R_i}^1$ for all R_i, hence $\frac{K_0 K_1}{K_0+K_1} \sim {R_i}^2$ for small R_i, and ~ ${R_i}^1$ for large R_i. Now, $x_{0b} = \varepsilon_b(R_i + L) + \varepsilon_{stalk} L_{stalk} \sim {R_i}^0$ for small R_i, and $\sim {R_i}^1$ for large R_i when L_{stalk} is fixed (i.e., $\sim {R_i}^0$) . Then Eq. S20 predicts $F_b \approx \frac{K_0 K_1}{K_0+K_1} x_{0b} \sim {R_i}^2$ and $\sigma_{max}^b \sim {R_i}^0$ for both small and large R_i. This explains the observed negligible change in attachment strength with nearly an order of magnitude increase in R_i with fixed L_{stalk}.

(3) Finally, $\frac{K_0 K_1}{K_0+K_1} x_{0b}$ in Eq. S20 increases linearly with elastic modulus, E, of the cups. This explains the observed increase in attachment strength by more than 2 times for all the cups tested when their E increased from 4 to 10 MPa.

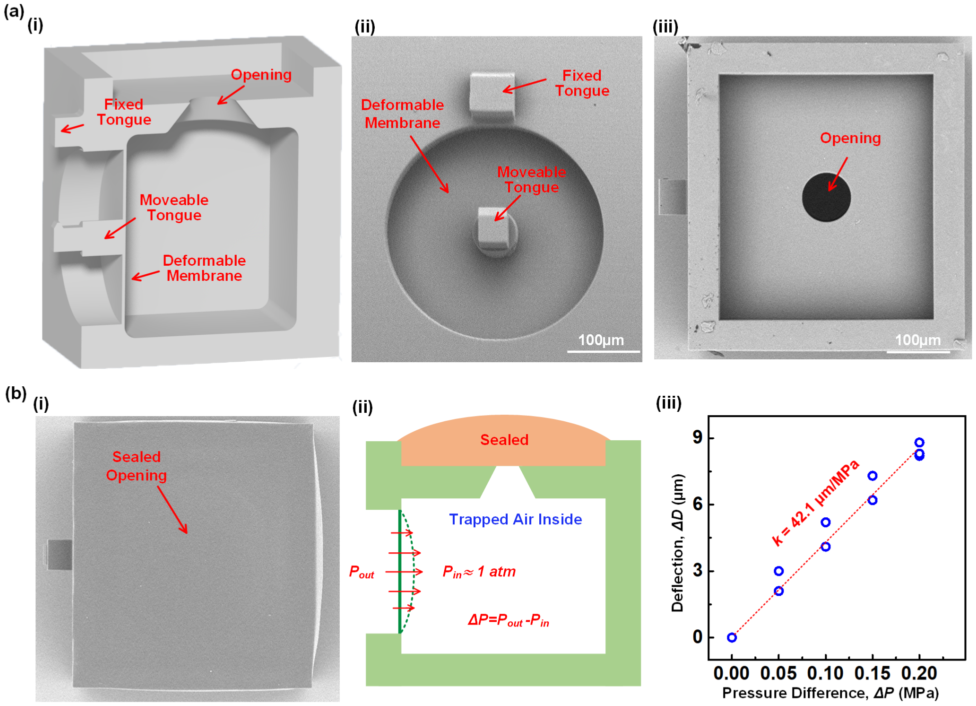

Figure S1. Design of the custom-made pressure sensor and calibration. (a) (i) Cross-section of the micro sensor (schematic); Scanning electron micrographs (SEM) showing (ii) side and (iii) top view with the 80 µm opening; **(b)** Sealed micro sensor for calibration: (i) SEM of the closed top; (ii) Schematic representation of the inward membrane deformation into the pressure chamber; (iii) Membrane deflection vs. pressure difference (blue circles). The red line represents linear fit.

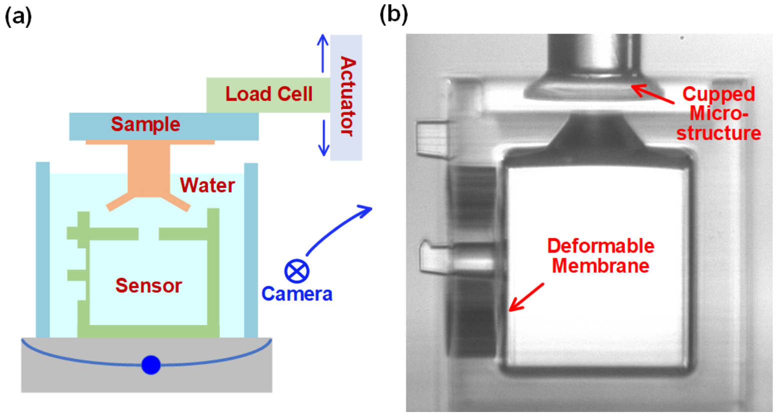

Figure S2. Under water adhesion testing. (a) Schematic of the custom-made test setup; **(b)** *in-situ* optical microscopy image of the micro-structure and built-in micro pressure sensor.

(a)

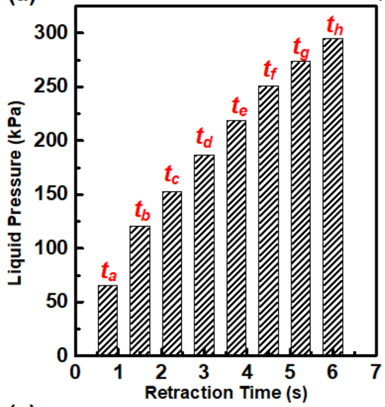

(b)

Moment	*ΔS* (μm)	*ΔP* (kPa)
t_a	2.8	66.5
t_b	5.1	121.1
t_c	6.4	152.0
t_d	7.9	187.6
t_e	9.2	218.5
t_f	10.6	251.8
t_g	11.5	273.1
t_h	12.4	294.5

(c)

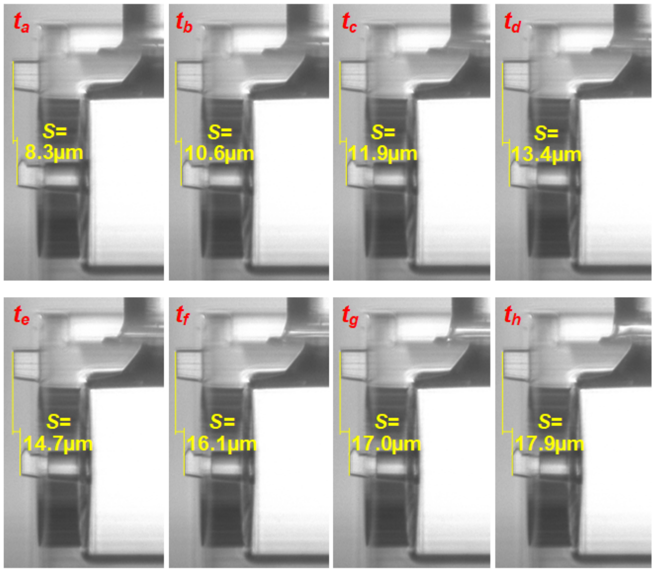

Figure S3. Membrane deflection. (a) Chart of liquid pressure changing with retraction of the cup; **(b)** The deflection of force sensing membrane and the corresponding liquid pressures at different time points of retraction; **(c)** *in-situ* images to monitor the membrane position at different retraction times. The initial distance between the membrane tongue and fixed reference tongue is 5.5 μm. Additional deflection due to suction is listed in (b). Image analysis was conducted with ImageJ.

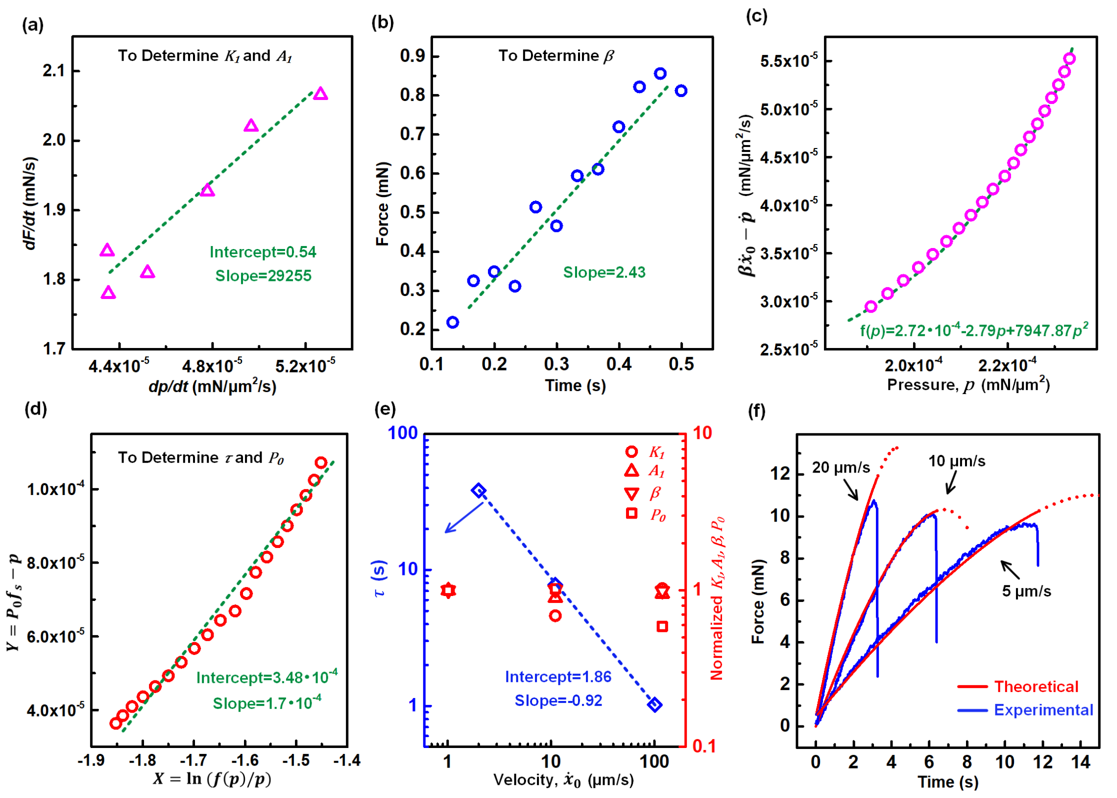

Figure S4. Identification of model parameters. (a) Rate of change of tested force and liquid pressure; **(b)** rate of force change at the beginning of retraction; **(c)** quadratic fitting between $\beta\dot{x}_0 - \dot{p}$ and pressure; **(d)** comparison between theoretical prediction and experimental data; **(e)** all the parameters P_0, β, A_1, K_1 and τ with retraction velocity change from 1 to 100 μm/s; **(f)** Evolution of force with time at different retraction speeds, model vs experiment.

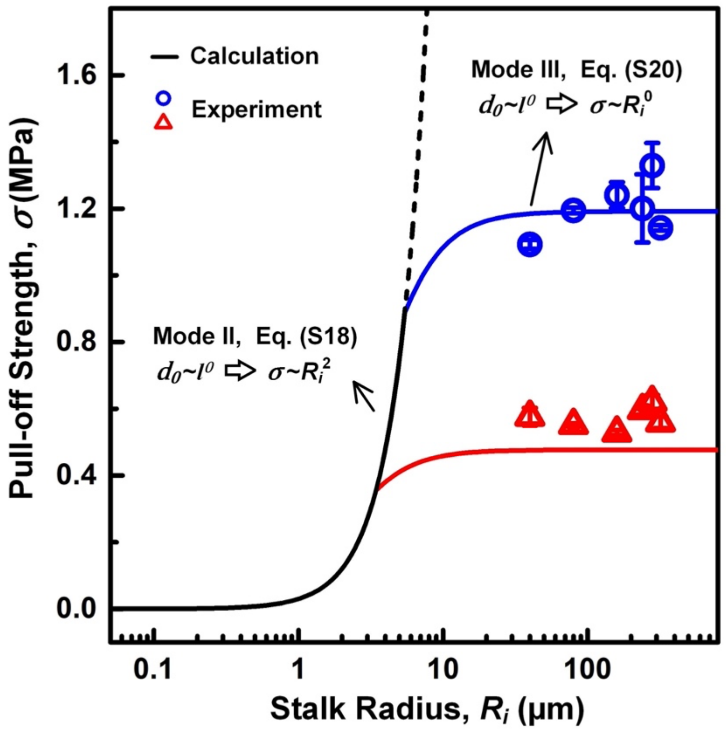

Figure S5. Experimental validation of scaling laws. Pull-off strengths are measured for cups with stalk radius, R_i, varying from 40-320 μm. Lip thickness and radius increased as $T = .1R_i$, R_1=1.2R_i respectively. Retraction speed, $\dot{x}_0 = 50$ μm/s for all cups. All cups detach in Mode III (buckling of lip). Detachment strength is independent of size, but it increases with cup elastic modulus, E, proportionately. E was increased from 4 MPa (red triangles) to 10 MPa (blue circles). These observations are predicted by the model based on the assumption of $d_0 \sim l^0$ representing lab conditions. Solid lines are prediction from model with d0=10nm, $\varepsilon_b = 5\%$, $\varepsilon_{stalk} = 10\%$.

Table S1. Calculated system parameters at different retraction velocities.

$\dot{x}_0$ (μm/s)	$K_1 \left(10^{-2}\frac{\text{mN}}{\mu\text{m}}\right)$	A_1 (10^4 μm^2)	$\beta \left(10^{-6}\frac{\text{mN}}{\mu\text{m}^3}\right)$	τ (s)	$P_0 \left(10^{-4}\frac{\text{mN}}{\mu\text{m}^2}\right)$
1	4.00	2.81	6.53	38.4	5.2
10	5.79	3.14	6.46	7.75	5.1
100	5.95	2.99	6.41	1.02	3.0
mean	5.24 ± 0.89	2.98 ± 0.13	6.47 ± 0.05	N/A	4.4 ± 0.1
